# Microbial Community Composition during a Bloom of Purple Bacteria in Intertidal Sediments in Vigo (Northwest Spain)

**DOI:** 10.1128/Spectrum.01238-21

**Published:** 2021-10-27

**Authors:** M. Froján, B. Arbones, J. L. Garrido, F. Rodríguez

**Affiliations:** a Department of Oceanography, Instituto de Investigaciones Marinas (IIM, CSIC), Vigo, Spain; b Department of Harmful Algae and Red Tides, Instituto Español de Oceanografia (IEO, CSIC), Vigo, Spain; University of Minnesota

**Keywords:** purple sulfur bacteria, microbial mat, *Chromatiaceae*, 16S rRNA, pigments

## Abstract

In summer 2019, a large, bright pink microbial mat was visible on top of macroalgal deposits in muddy sediments of an urban beach (Playa do Adro, Vigo). In order to characterize the dominant organisms in these colored mats, results from microscopic observations, photosynthetic pigments, and molecular analysis were gathered. Light microscopy examination revealed pinkish microbial aggregates with minor contributions of larger protists and cyanobacteria. High-performance liquid chromatography (HPLC) pigment analysis documented the dominance of bacteriochlorophyll *a* and carotenoids whose spectra were compatible with those described in photosynthetic purple bacteria. 16S rRNA gene amplicon sequencing confirmed that the vast majority of reads belonged to *Proteobacteria* (73.5%), and among them, nearly 88% of those reads belonged to purple sulfur bacteria (*Gammaproteobacteria*). A single family, *Chromatiaceae*, constituted the bulk of this assemblage, including the genera *Thiohalocapsa* (32%), *Marichromatium* (12.5%), *Phaeochromatium* (5%), and *Halocromatium* (2%) as main contributors. Nonetheless, a considerable number of sequences could not be assigned to a particular genus, stressing the large biological diversity in these microbial mats and the potential presence of novel taxa of purple sulfur bacteria.

**IMPORTANCE** Urban beaches are valuable recreational areas particularly vulnerable to human disturbance. In these areas, the intertidal sediments harbor a diverse community of microorganisms, including virus, bacteria, fungi, and protozoa. In this sense, pollution events can introduce pathogenic allochthonous microbes which may constitute a human health risk. Visual and sensory observations, such as a weird color or bad smell, are usually appreciated as a warning by beachgoers and authorities, as indeed was the case at do Adro beach in 2019. The observed proliferation seems to be common in summertime, but its dimension alerted beachgoers and media. The obtained results allowed for the identification of purple sulfur bacteria as responsible for the pink-violet top layer staining the intertidal zone. These blooms have never been associated with public health risks. Beyond solving the sanitary concern, other important findings were its diversity and large proportion of novel taxa, illustrating the complexity of these ecosystems.

## INTRODUCTION

Microbial mats in intertidal sediments integrate diverse communities layered vertically following their physiology (1, 2). The exposed sediments are usually dominated by cyanobacteria and diatom assemblages, while anoxygenic photolithotrophs (purple and green bacteria) thrive beneath. Down these layers, in anaerobic conditions, the metabolic activity of sulfate-reducing bacteria generates sulfides that diffuse upwards, where they end up being used by some anoxygenic phototrophic bacteria (3).

Phototrophic purple bacteria are a diverse phylogenetic and functional group represented widely in aquatic and terrestrial ecosystems (4). Within them, the nonsulfur (*Alphaproteobacteria* and *Betaproteobacteria*) and sulfur bacteria (*Gammaproteobacteria*) can be distinguished. These organisms can grow photoautotrophically at anoxic or suboxic conditions, whereas at higher oxygen levels, they can downregulate photosynthesis to exploit organic carbon sources, as well (5).

Purple nonsulfur bacteria thrive either phototrophically or chemotrophically in darkness. In contrast, purple sulfur bacteria (PSB) are primarily phototrophic and their populations rely on sulfide and light availability for their growth. Thus, in contrast to cyanobacteria, PSB perform anaerobic and anoxygenic photosynthesis (5). These organisms, together with other aerobic anoxygenic phototrophs, display a wide arrange of colors associated with carotenoids. These compounds may play photosynthetic and nonphotosynthetic functions, but the main photosynthetic pigments capable of absorbing light energy are the bacteriochlorophylls (BChl) (6).

In muddy sediments, PSB are known to proliferate at a depth of about 1 to 2 mm; however, they can proliferate on sandy sediments enriched in organic matter during summer, forming large colored mats. The occurrence of these blooms has been recorded in both pristine and anthropogenically influenced environments (7).

In recent decades, several authors have described the species composition of microbial mats in anoxic sediments, based on chemical and molecular analyses (8–12), that cooccur with oxygenic phototrophic organisms like cyanobacteria and diatoms that tend to dominate illuminated benthic environments (13). However, available studies show that purple bacteria, sulfate-reducing bacteria (*Deltaproteobacteria*), *Bacteroidetes*, *Cyanobacteria*, and *Actinobacteria* can also be prominent bacterial divisions in coastal microbial mats (see reference 2 and references therein).

During summer 2019, a large, bright pink microbial mat was visible on an urban beach at Bouzas (Vigo), accompanied by a smell of rotten eggs, like that of sulfur. The location is usually crowded by beachgoers during summer, and local people confirmed that these pink mats were recurrent for the season and not exceptional by any means. Also, to our knowledge, they have not been reported in another time of the year. Despite that, no previous studies have ever been performed to identify the proliferating organisms. In the present study, the microbial community composition and pigment/optical properties of the organisms responsible for the pink mats in Vigo were obtained by means of 16S rRNA gene amplicon sequencing, spectrophotometric analyses, and high-performance liquid chromatography (HPLC) analyses.

## RESULTS

### Microscopic observations.

**FIG 1:**
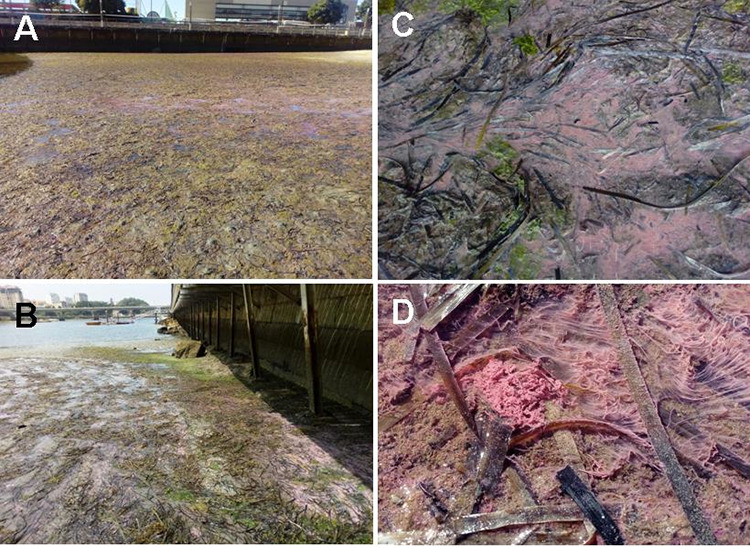
Pink microbial mats in do Adro beach, in Bouzas (Vigo). (A, B) General overview of the muddy sediments near the shore during low tide. (C, D) Close-up of pink mats entangled with *Zostera* leaves.

**FIG 2:**
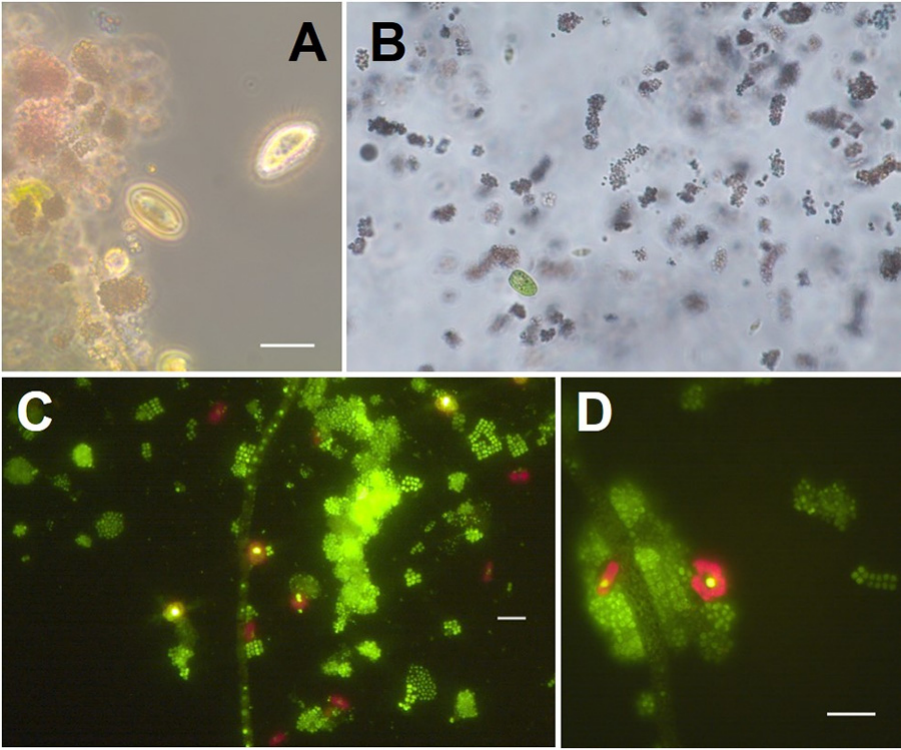
Microscopic images of samples collected from the pink mats. (A, B) Light microscopy images showing microcolonies of small-celled sulfur purple bacteria with pinkish aspect along with other mat members like benthic diatoms, ciliates, or free-living flagellates. (C, D) Epifluorescence micrographs of SYBR green-stained samples showing different morphotypes of PSB aggregates, including dense rounded colonies or colonies with a scattered cell distribution and also squarish colonies with cells disposed in parallel rows. Red autofluorescence indicates the presence of chloroplasts. Scale bars: 10 μm.

Pink aggregates in the studied microbial mats at Playa do Adro, in Vigo (Fig. 1), were first observed under light microscopy (LM) and epifluorescence as detailed in Figure 2. Dense colonies were observed with a light pinkish aspect, entangled with organic debris and other prokaryotic and eukaryotic organisms. Among these, colonies of cyanobacteria (e.g., *Merismopedia* and *Oscillatoria*), pennate diatoms, green flagellates, and dinoflagellates were observed, together with ciliates (e.g., *Paramecium*, among others).

### Spectral properties and pigment analyses.

**FIG 3:**
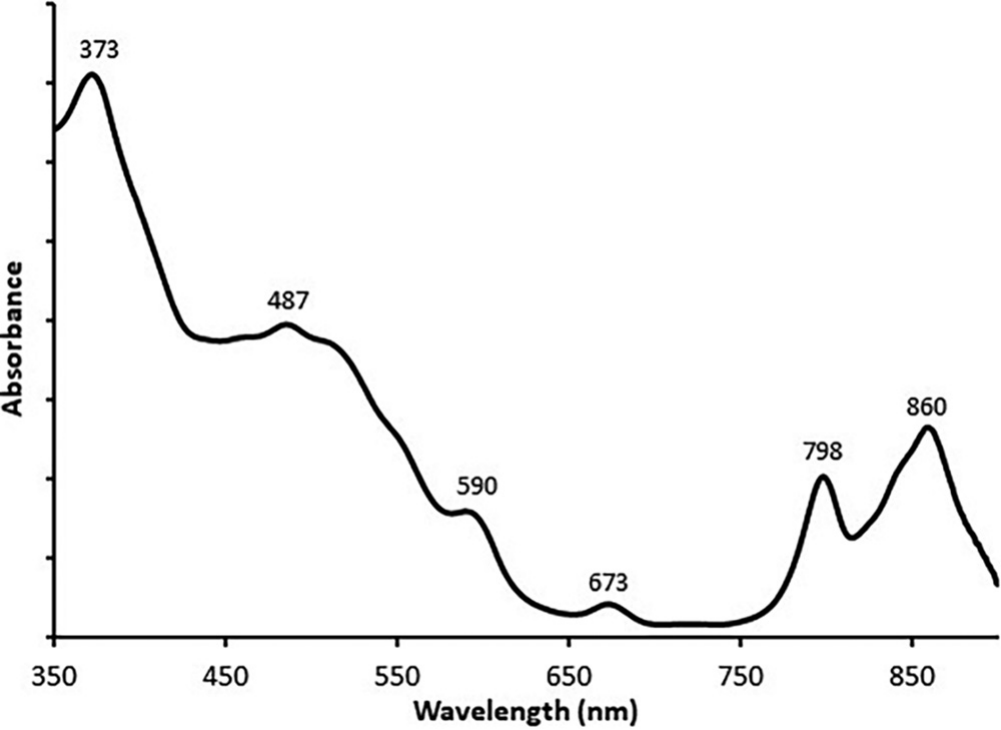
Visible spectrum of the cell extract from pink mats, in phosphate buffer.

The visible spectrum of the cell extract in phosphate buffer (Fig. 3) shows major peaks indicative of the predominance in the microbial mat of organisms containing Bchl *a*-incorporating protein-pigment complexes: 373 nm (Soret band), 590 nm (Q_x_ band), and 798 and 860 nm (Q_y_ bands). The absorbance between 450 and 550 nm arises mainly from carotenoids. The band at 673 nm could be attributed to chlorophyll (Chl) *a* complexes of eukaryotic photosynthetic organisms also occurring in the mat.

**FIG 4:**
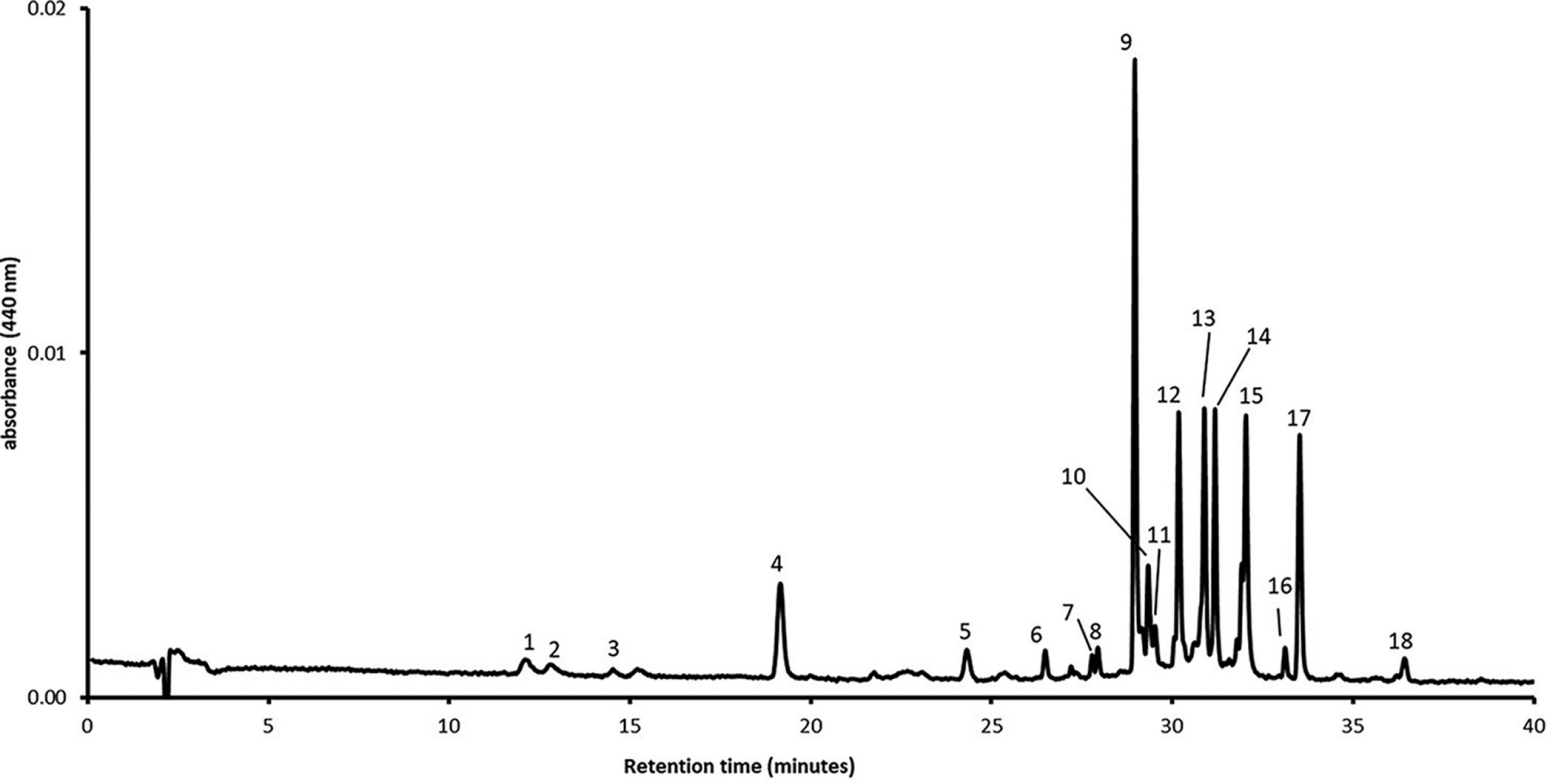
HPLC pigment analysis of a selected representative sample from the pink mats.

Pigment analysis by HPLC allowed the detection of minor but qualitatively significant amounts of marine phytoplankton pigments and the presence of Bchl *a* and carotenoids whose spectra are compatible with those described in photosynthetic purple bacteria (Fig. 4 and Table 1).

**TABLE 1:** Identification of pigments detected using HPLC analysis; wavelengths in parentheses denote shoulders

Peak	Retention time (min)	Maxima in eluant (nm)	Pigment
1	12.09	452, 583, 633	Chlorophyll *c*_2_
2	12.78	448, 580, 631	Chlorophyll *c*_1_
3	14.52	473	Peridinin
4	19.16	449	Fucoxanthin
5	24.31	417, 441, 470	Diadinoxanthin
6	26.48	430, 453, 483	Alloxanthin
7	27.78	(426), 453, 478	Zeaxanthin
8	27.94	(422), 446, 475	Lutein
9	28.96	490 (518)	Unknown keto-carotenoid (tentative)
10	29.33	485	Unknown keto-carotenoid (tentative)
11	29.52	469, 496, 529	Hydroxyspirilloxanthin (tentative)
12	30.18	486	Unknown keto-carotenoid (tentative)
13	30.89	469, 496, 529	Spirilloxanthin (tentative)
14	31.18	448, 473, 504	Rhodopin (tentative)
15	32.04	363, 602, 770	Bacteriochlorophyll *a*
16	33.12	448, 474, 504	Lycopene
17	33.52	431, 617, 662	Chlorophyll *a*
18	36.43	(426), 452, 477	β,β Carotene

Apart from Chl *a*, present in most prokaryotic and eukaryotic phytoplankton organisms, the occurrence of Chls *c*_1_ and *c*_2_ and fucoxanthin can be linked to diatoms, peridinin is unequivocally associated with dinoflagellates, alloxanthin suggests the presence of cryptophytes, and the isomeric xanthophyll pair zeaxanthin and lutein points to the presence of members of the green microalgae lineage.

The lack of standards of the characteristic carotenoids of purple bacteria prevented certainty in their identification, except in the case of lycopene, whose spectrum and chromatographic behavior exactly matched those of lycopene extracted and purified in our laboratory from tomato peel. Peak 14 was tentatively identified as rhodopin due to the coincidence of its spectrum with that of lycopene (peak 16), with whom it shares a chromophore of 11 conjugated double bonds.

The bathochromic shift of the maxima of the spectrum and its fine structure indicates a chromophore of 13 double bonds for both peaks 13 and 11, which, together with their chromatographic behaviors, suggests that their tentative identities may be spirilloxanthin and hydroxyspirilloxanthin, respectively. A detailed analysis of the spectra of the remaining peaks of the chromatogram, even the minor ones, did not allow us to find spectra attributable to rhodovibrin or its derivatives (12 conjugated double bonds). Finally, three relevant peaks in the chromatogram (namely, peaks 9, 11, and 12) showed a rounded spectrum, lacking fine structure, characteristic of keto-carotenoids.

### Microbial community composition.

**FIG 5:**
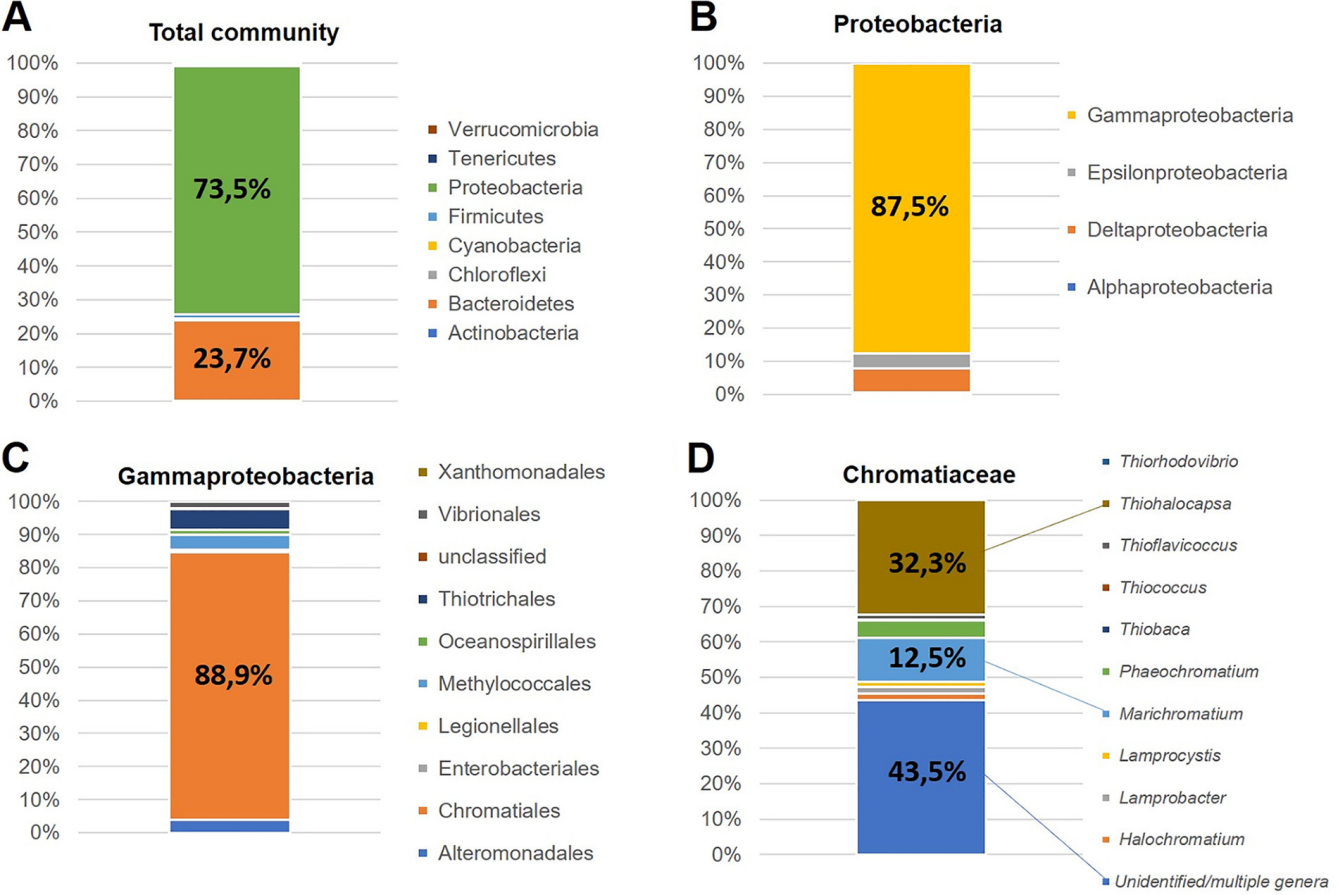
Genetic analysis of a sample from the pink mats. (A) Total community 16S rRNA reads of bacterial phyla. (B) Bacterial classes within *Proteobacteria*, the dominant phylum. (C) Bacterial orders within *Gammaproteobacteria*. (D) Bacterial genera within family *Chromatiaceae*.

16S rRNA gene amplicon sequencing retrieved 69,712 reads, and 100% of sequence tags could be assigned at phylum and class level. The vast majority of these belonged to *Proteobacteria* (73.5%) and *Bacteroidetes* (23.7%), while a mixed assemblage of *Firmicutes*, *Cyanobacteria*, *Tenericutes*, and *Actinobacteria*, among others, accounted for <3% of the phyla (Fig. 5A).

Regarding *Proteobacteria*, nearly 88% of reads belonged to *Gammaproteobacteria*, with sulfate-reducing bacteria (*Deltaproteobacteria*) and *Epsilonproteobacteria* contributing 7% and 5% of the community (Fig. 5B). These *Deltaproteobacteria* were constituted almost totally by *Desulfobacterales*, dominated by the *Desulfobulbaceae* family (88%).

Over 99% of reads were assigned at the order level. The most important component of the microbial community, *Gammaproteobacteria*, belonged primarily to the order *Chromatiales* (80%; Fig. 5C), followed by *Thiotrichales* (*Piscirickettsiaceae*) and *Methylococcales* (*Methylococcaceae*), with 6% and 4% of reads. The rest, <10%, included several orders, mainly *Alteromonadales*, *Oceanospirillales*, and *Vibrionales*.

Finally, a single family of PSB, *Chromatiaceae*, constituted the bulk of *Chromatiales*, including the genera *Thiohalocapsa* (32%), *Marichromatium* (12.5%), *Phaeochromatium* (5%), and *Halocromatium* (2%) as the main contributors identified. Nevertheless, a significant proportion of reads (43.5%) could not be assigned to any (or a single) genus (Fig. 5D).

## DISCUSSION

Microbial communities in intertidal coastal mats are exposed to large variations in environmental conditions (e.g., salinity and temperature) where a transition between oxic and anoxic conditions develops along steep gradients of oxygen and sulfide (1). During summer, the organic matter available from the macroalgal deposits together with increased irradiance and temperature conditions set up the basis for the development of multicolored layers of several groups, including *Cyanobacteria*, benthic diatoms, and anoxygenic phototrophic bacteria. These can include green and purple sulfur bacteria, as well as white layers of sulfate-oxidizing bacteria, followed downwards by sulfate-reducing bacteria in the black mud produced after the precipitation of iron sulfides (1).

The colored microbial mats observed in this study have never been characterized in depth, to our knowledge, in Galicia, despite the fact that they are likely found in other intertidal areas with similar hydrodynamic and sediment characteristics inside the Rías. The first approximation to the organisms under study was given by its pink-violet color and by the fact that this color was completely extracted into acetone solutions, which suggested its carotenoid nature, supporting that the organisms could be purple bacteria (14).

The visible spectrum of crude extracts of photosynthetic membranes of the microbial mat under study (Fig. 3) resembles similar spectra described for phototrophic purple bacteria (3, 15). The characteristic shape of this spectrum in purple bacteria is usually dominated by the contribution of the peripheral light-harvesting antenna (LH2) that provides two absorption bands around 800 and 850 nm (798 and 861 nm in this study) that correspond to bacteriochlorophylls arranged into two different rings (B800 and B850) in the pigment-protein complex (16–18).

Two main carotenoid biosynthesis pathways have been proposed in purple bacteria, the spirilloxanthin pathway (normal spirilloxanthin, unusual spirilloxanthin, spheroidene, and carotenal pathways) and the okenone pathway (okenone and *R.g.* keto-carotenoid pathways) (14). The presence of lycopene and Bchl *a* and the tentative identification of spirilloxanthin and rhodopin, as well as three unknown keto-carotenoids, suggest the occurrence of a variety of organisms in which both biosynthetic pathways are being expressed and support the hypothesis of the presence of members of the *Chromatiaceae* family (i.e., *Halochromatium*, *Marichromatium*, *Thiohalocapsa*, etc.) in which both pathways are frequent (14).

Together with the mat, typical phytoplankton organisms in these coastal waters were evident both by the presence of marker pigments (19) in the chromatogram (e.g., fucoxanthin, Chls *c*, peridinin, alloxanthin) and by the appearance in the spectrum of the crude extract in phosphate buffer of a band at 673 nm that has been described in the spectra of diatom thylakoid membranes (20).

Furthermore, 16S rRNA gene sequencing from the pink microbial mat in Vigo confirmed the dominance of purple sulfur bacteria in the sampled surface layer. The results in the present study correspond with previous findings on the Orkney Islands (21, 22) and in Roscoff Aber Bay (1), in beaches where high levels of organic matter are seasonally observed due to decomposition of macroalgae. This also seems to be the situation in playa do Adro, where a dense mixture of leaves from aquatic plants (Zostera marina) together with green and brown macroalgae were found covering its shore in low tide.

Former authors have reviewed the most important PSB in these environments (1, 2, 23, 24), mentioning some genera and species from the family *Chromatiaceae*, like Thiocapsa roseopersicina, Thiocystis violacea, and Allochromatium vinosum. In particular, *Allochromatium* spp. and *Marichromatium* spp. are commonly recorded and sometimes can be dominant. For example, in the pink layer of microbial mats of the Ebro Delta, Spain (8), there were retrieved several genera of *Chromatiaceae*, like *Thiospirillum*, *Marichromatium*, *Thiocapsa*, and *Chloroflexus*.

Thus, our molecular results were in agreement with these previous studies, indicating the dominance of PSB and the family *Chromatiaceae*, which can account for a major fraction of the photosynthetic biomass in the pink layers of microbial mats (8).

The detection of a considerable number of reads in the present study that could not be assigned to a single genus calls the attention toward the large biological diversity found in these coastal microbial mats and the potential presence of novel taxa not related to any known PSB.

## MATERIALS AND METHODS

### Study area and sample collection.

On 3 July 2019, local press reported a warning raised by some citizens about the occurrence of large bright pink mats in an urban beach (do Adro), located in a small inlet in Bouzas (42°13′32″N, −8°45′13″W; Vigo). The beach has a smooth slope, with sandy/muddy sediments, in shallow protected waters with southwestern orientation toward the Ría de Vigo (Fig. 1A and B). The estimated area for the pink mats would range from 600 to 900 m^2^ from pictures taken on the site and using Google Earth tool for surface measurement. In this sense, a pink microbial mat was apparent on that site (accessed 13 Aug 2021), but imagery date information is not available in Google Earth web version.

On July 7, surface samples were obtained at this site. Sediments contained extensive organic deposits, mainly Zostera marina leaves and green macroalgae (filamentous and *Ulva* sp.), covered by dense pink mats of filamentous aspect around 1 to 2 cm thick (Fig. 1C and D). Samples that contained only bright pink microbial mats were chosen by naked eye in order to avoid as much interference from sediments, macroalgae, and *Zostera* debris as possible. No information about the vertical layering of benthic communities was recorded.

Floating pink filaments were collected directly by submerging 50-ml Falcon tubes and 25-ml glass vials in the top seawater layer containing pink mats. Overall, 10 samples were collected from small ponds that formed *in situ* during low tide for further analysis (see sections below). Samples were collected around the middle part of the exposed pink mat, separated 2 to 5 m each within an area of ∼100 m^2^. Other microorganisms from seawater or benthos would be expected, as no sample processing was done before the analyses, but in minor amount.

### Light microscopy.

Several *in vivo* samples were inspected in an epifluorescence microscope (Leica DMLA, Wetzlar, Germany) equipped with a UV light source and an AxioCam HRc (Carl Zeiss, Jena, Germany) digital camera. Subsamples of 10 ml were directly stained with 4 μl of SYBR green dye for 10 minutes in the dark. Subsequently, these subsamples were filtered through 0.2 μm black Millipore-Isopore filters, and were visualized on a Nikon Eclipse TE200-S epifluorescence microscope, illuminating the filters with blue light.

### Spectrophotometric measurements and HPLC pigment analyses.

Six samples were allowed to settle in the vials in the dark at room temperature. In each of them, the supernatant seawater was carefully removed and the resulting thick pink-purple slurry was used for pigment analysis. Three samples were employed for the spectral analysis of the photosynthetic membranes: 1 ml of each slurry was suspended in 9 ml of 50 mM phosphate buffer (pH 7.2) by sonication, as described in reference 12. The samples were pooled and the resulting solution was scanned between 350 and 900 nm. For pigment analysis by HPLC, 1 ml of slurry from each of the remaining samples was added with 9 ml of acetone and extracted until complete discoloration as described in reference 25. The three pigment extracts were analyzed separately following reference 26.

### Genetic analysis.

Small aliquots of pink aggregates (∼25 ml) for molecular analyses were centrifuged on 50-ml Falcon tubes (7,000 × *g*, 10 min). After the supernatants were discarded, pellets were transferred to Eppendorf tubes and frozen at −40°C until further analysis. A single sample was selected to study the bacterial composition using 16S rRNA gene amplification, sequencing, and analysis.

### Library construction and quantification.

The 16S rRNA gene was amplified with 16S ion metagenomics kit (Life Technologies), which is designed for rapid analysis of polybacterial samples using Ion Torrent sequencing technology. This kit uses two primer pools that selectively amplify seven hypervariable regions of bacterial 16S rRNA gene (V2, V4, V8, and V3, V6, V7, V9). Each reaction contained 2 ng of template DNA. After PCR, the amplicons were equally combined and quantified using the Agilent 2100 Bioanalyzer. Libraries were carried out using the Ion Plus library kit for AB Library Builder System (ThermoFisher Scientific) following the library preparation protocol for short amplicons. A total of 300 ng of amplified DNA was used for library preparation. The resulting DNA libraries were subsequently quantified by qPCR using the Ion universal library quantitation kit (ThermoFisher Scientific), following manufacturer’s instructions, to calculate the dilution factor of the library to get a final concentration of 40 pmol.

### Template preparation and sequencing.

Emulsion PCR and chip loading were performed in the Ion Chef instrument (ThermoFisher Scientific) following the instructions of the manufacturer. Finally, sequencing was performed on the Ion Personal Genome Machine (PGM) using the Ion Hi-Q sequencing view kit (400 bp) and 316 V2 BC chip. After sequencing, base calling and run demultiplexing were performed by Torrent Suite Software version 5.2.2 (ThermoFisher Scientific) with default parameters. The number of reads was 239,437 with a mean read length of 193 bp. FileExporter version 5.2.0.0 (ThermoFisher Scientific) was used to generate BAM files. The BAM files were analyzed using the 16S Metagenomics workflow v5.4 of the Ion Reporter Software. This enables the rapid identification of microbes present in complex polybacterial research samples using both curated Greengenes (27) and premium curated MicroSEQ ID 16S rRNA (Life Technologies) reference databases.

### Data availability.

Data were deposited in the NCBI SRA database under accession PRJNA759770.
